# Collagen and Beyond: A Comprehensive Comparison of Human ECM Properties Derived from Various Tissue Sources for Regenerative Medicine Applications

**DOI:** 10.3390/jfb14070363

**Published:** 2023-07-11

**Authors:** Nashaita Y. Patrawalla, Nilabh S. Kajave, Mohammad Z. Albanna, Vipuil Kishore

**Affiliations:** 1 Department of Biomedical and Chemical Engineering and Sciences, Florida Institute of Technology, Melbourne, FL 32901, USA; 2 Humabiologics® Inc., Phoenix, AZ 85034, USA; 3 Department of General Surgery, Atrium Health Wake Forest Baptist, Winston-Salem, NC 27101, USA

**Keywords:** collagen, ECM, hydrogels, decellularization, human tissue, mesenchymal stem cells

## Abstract

Collagen, along with proteoglycans, glycosaminoglycans, glycoproteins, and various growth factors, forms the extracellular matrix (ECM) and contributes to the complexity and diversity of different tissues. Herein, we compared the physicochemical and biological properties of ECM hydrogels derived from four different human tissues: skin, bone, fat, and birth. Pure human collagen type I hydrogels were used as control. Physical characterization of ECM hydrogels and assessment of cell response of cord-tissue mesenchymal stem cells (CMSCs) were performed. Decellularization efficiency was found to be >90% for all ECM. Hydroxyproline quantification assay showed that collagen content in birth ECM was comparable to collagen control and significantly greater than other sources of ECM. Sodium dodecyl-sulfate polyacrylamide gel electrophoresis (SDS-PAGE) analysis showed the presence of γ, β, α_1_ and α_2_ collagen chains in all ECMs. Gelation kinetics of ECM hydrogels was significantly slower than collagen control. Compressive modulus of skin ECM was the highest and birth ECM was the lowest. Skin and birth ECM hydrogels were more stable than bone and fat ECM hydrogels. CMSCs encapsulated in birth ECM hydrogels exhibited the highest metabolic activity. Rheological characterization revealed that all ECM-derived inks exhibited shear thinning properties, and skin-derived ECM inks were most suitable for extrusion-based bioprinting for the concentration and printing conditions used in this study. Overall, results demonstrate that the physicochemical and biological properties of ECM hydrogels vary significantly depending on the tissue source. Therefore, careful selection of tissue source is important for development of ECM-based biomimetic tissue constructs for regenerative medicine applications.

## 1. Introduction

Collagen is the most ubiquitous protein found in the human body [1]. Of the 28 different types of collagen, collagen type I is widely used in tissue engineering and regenerative medicine applications owing to its biocompatibility, biodegradability, and availability of cell adhesion sites [2]. Additionally, ease of processing enabled by the unique ability of collagen type I monomers to self-assemble and undergo fibrillogenesis is a significant advantage to form cell-laden hydrogel-based scaffolds [3]. Over the last three decades, a plethora of different physical and chemical crosslinking schemes as well as biofabrication methods have been developed to yield collagen scaffolds that mimic the physicochemical properties of native tissues [4,5,6,7]. However, in biomimetic approaches, single protein systems do not recapitulate the physical and biological complexities of the native tissue extracellular matrix (ECM). 

Tissue ECM is mainly composed of collagen, along with proteoglycans, glycosaminoglycans (GAGs), glycoproteins, and various growth factors [8]. The complexity and diversity in human tissues can be attributed to the unique compositional and structural properties of the ECM. For example, apart from collagen, the ECM in fat tissue is rich in laminin while the major component in skin ECM is elastin [9,10]. Prior work comparing the compositional make up of bone-marrow and fat ECM reported that while both tissues primarily composed of collagen type VI and fibronectin, the type and amount of glycoproteins and proteoglycans differ [11]. The presence of non-collagenous proteins complements the in vivo biophysical microenvironment necessary for cell adhesion, proliferation, and differentiation [12]. For example, laminin readily forms a network with collagen type IV and has been shown to promote cell migration and growth [13,14]. On the other hand, fibronectin crosslinks with collagen type I and modulates ECM organization as well as cell cytoskeletal arrangement [15,16] and elastin is associated with mechanical integrity of the tissues [17,18].

Animal welfare and the 3Rs principle, referring to replacement, reduction, and refinement of animals used in research, teaching, testing, and exhibition, have recently promoted the use of human-derived biomaterials in regenerative medicine applications as a valuable alternative to animal-derived biomaterials. In addition, potential infectious disease transmission and immunological reactions remain among the major concerns of using animal-derived biomaterials in developing human regenerative therapies. Several studies have demonstrated that the ultrastructure of ECM composition varies significantly from and within animal species [19,20] and between human and animals [21]. Recently, Schmitt et al. demonstrated that application of low concentration xenogenic collagen bioinks failed to yield stable bioprinted structures compared to human bioinks [22]. In a separate study, Bedell et al. compared human and porcine gelatin methacryloyl (GelMA) hydrogels and showed that human GelMA had higher mechanical properties, longer gel stability, higher cell viability, and more enhanced osteogenic differentiation of mesenchymal stem cells (MSCs) [23]. Other alternatives to animal-derived biomaterials include recombinant human proteins and/or gene editing, which are costly, time consuming, and against the 3R principle [21]. Thus, there is a pressing need to encourage researchers to utilize clinically relevant human biomaterials derived from native human tissues.

Recreating the complex ECM microenvironment is critical to provide the essential biophysical and biochemical cues to stimulate tissue-specific cell response and function. Towards this goal, commercially available basement membrane matrix, such as Matrigel^®^, PermaCol^TM^ and Veritas^®^, were routinely used in prior studies to mimic human tissue niche, but these are associated with significant drawbacks that include the presence of xenogeneic contaminants and risk of pathogenic transmission [24]. Multicomponent ECM hydrogels derived from decellularized human tissues provide a promising alternative to single component collagen biomaterial by preserving the complex native compositional niche [25]. Decellularized tissue ECM scaffolds have been shown to support cell adhesion, proliferation, and differentiation, and can be beneficial for a range of different biomedical applications, including 3D cell culture, organoids, and bioprinting [26,27]. For example, prior work has shown that the mechanical properties of decellularized dermal-derived ECM scaffolds were comparable to native skin tissue [28]. In a separate study, Yang et al. demonstrated enhanced tenogenic differentiation of adipose stem cells cultured on tendon-derived ECM scaffolds compared to cells cultured on pure-collagen scaffolds [29]. MSCs cultured on two different ECMs were shown to respond to tissue-specific cues and differentiate to osteogenic or adipogenic lineage when cultured on bone-marrow or adipose-derived ECMs, respectively [11].

Clearly, inherent differences in the compositional makeup of the ECM derived from different tissues can significantly impact scaffold properties and cell function. However, a systematic study that entails a direct comparison of the physicochemical and biological properties of scaffolds synthesized using decellularized ECM from multiple different human-derived tissues is lacking. The current study is the first attempt to compare ECM hydrogels derived from four different decellularized human tissues: skin ECM, bone ECM, fat ECM, and birth ECM. Pure collagen type I hydrogels were used as control. Physical characterization of tissue ECM hydrogels entailed assessment of gelation kinetics, mechanical properties, stability, and surface microstructure. Cell viability and metabolic activity assays were carried out to assess the effects of different tissue ECM on cord-tissue mesenchymal stem cell (CMSC) response. Additionally, this work is also the first time that the rheological properties and printability of ECM inks derived from different tissues has been examined. The outcomes of this work can be highly beneficial to enable judicious selection of ECM-based biomaterials for tissue-specific biomedical applications.

## 2. Materials and Methods

### 2.1. Materials

Quant-iT Picogreen dsDNA kit (Cat# P11496), live-dead assay kit (Cat# L32250), and alamarBlue^TM^ (Cat# DAL1025) were purchased from Invitrogen (Carlsbad, CA, USA). The electrophoresis system was purchased from Bio-Rad (Hercules, CA, USA). Hydroxyproline assay kit was purchased from Abcam (Cat# ab222941/K226) (Cambridge, UK). CMSCs were isolated in-house from the cord tissues. All other chemicals and reagents were purchased from Fisher Scientific (Waltham, CA, USA), unless stated otherwise.

### 2.2. Extraction of Human Tissue ECM and DNA Quantification

Cadaveric donor human tissues were obtained from a US-accredited tissue bank. ECM extraction and solubilization protocols were developed by Humabiologics^®^, Inc by employing a proprietary combination of physical, chemical, and enzymatic approaches. To evaluate the efficacy of the decellularization process, the remnant DNA from decellularized tissue ECMs was extracted using proteinase K solution at 37 °C for 24 h and quantified using Quant-iT Picogreen dsDNA kit (N = 3/group). Percent DNA removal was calculated by determining the total DNA amount in human tissues prior to decellularization using the same process. Briefly, 100 µL of solubilized tissue ECM before and after decellularization from each tissue source and equal volume of dsDNA reagent was added into individual wells of a 96-well plate. The plate was incubated at room temperature for 5 min by covering it with aluminum foil to protect it from light. Following this, fluorescence was measured at an excitation wavelength of 480 nm and emission wavelength of 520 nm using M2 Spectramax plate reader (Molecular Devices, San Jose, CA, USA). DNA concentration in each sample was calculated by generating a standard curve with known concentrations of DNA.

### 2.3. Quantification of Hydroxyproline in Tissue ECM Solutions

To quantify the collagen content in tissue ECM solutions, the amount of hydroxyproline (Hyp) was measured using a hydroxyproline assay kit. Briefly, tissue ECM solutions (N = 3/group) were mixed with equal volumes of 10N NaOH and hydrolyzed at 120 °C for 1 h. Following this, the mixture was cooled on ice, and neutralized with 10N HCl. The mixture was then centrifuged at 8000× *g* for 5 min and the supernatant was collected in a new tube. The sample hydrolysate was then evaporated by incubating the plate at 65 °C after transferring 10 µL aliquots from each tube to a 96-well plate. Further, 100 µL of the oxidation reagent mix was added to each well, and the plate was incubated at room temperature for 20 min. Next, 50 µL of developer solution was added to each well, and the plate was incubated at 37 °C for 5 min. The DMAB concentrate solution was then added to each well, and the plate was incubated at 65 °C for 45 min. Following this, the absorbance was measured in a M2 Spectramax plate reader (Molecular Devices) at 560 nm. A standard curve was obtained by measuring the absorbance of known Hyp concentrations ranging from 0–0.1 µg/µL. The Hyp content in the tissue ECMs was calculated by using Equation (1), where B is the amount of hydrolyzed Hyp (µg) calculated from the standard curve, V is the sample volume (µL) added to the well, and D is the dilution factor of the sample to fit in the range of the standard curve.

(1) $$Hyp\;Concentration=\left(\frac{B}{V}\right)*D$$

### 2.4. Sodium Dodecyl Sulfate-Polyacrylamide Gel Electrophoresis (SDS-PAGE)

SDS-PAGE was performed to characterize the purity of different tissue ECMs (N = 3) using 8% separating gels and 4% stacking gels. Different tissue ECM solutions (6 mg/mL), collagen type I (6 mg/mL), and control (15 mM HCl) were mixed individually in 1.5 M Tris-HCl buffer containing SDS and β-mercaptoethanol and heated at 100 °C for 5 min. Following this, 10–15 µL of sample solution was loaded and electrophoresed at 120 V for 2 h. Gels were then stained with 0.25% Coomassie Blue R-250 solution (Cat# 20278) (40% distilled water, 50% ethanol, and 10% glacial acetic acid) for 35–40 min and de-stained in ethanol/acetic acid solution until the resulting bands were distinct from the background. High magnification images were taken using a triple-lens camera.

### 2.5. Synthesis of Human Tissue ECM Hydrogels

Solubilized ECM solutions were subjected to an in-house gelation protocol to form ECM hydrogels. Briefly, tissue-specific ECM hydrogels were prepared by mixing nine parts of acid-soluble ECM solution (6 mg/mL) with one part of 10x PBS (pH 7.4). Following this, the mixture was incubated at 37 °C for 60 min to induce fibrillogenesis and gelation. Pure human skin-derived collagen type I was used as control.

### 2.6. Assessment of Gelation Kinetics of Human Tissue ECM Solutions

Gelation kinetics assay was performed to determine the polymerization rate of ECM solutions derived from different tissue sources. Briefly, 100 µL of chilled neutralized ECM solution was casted into individual wells of a 96-well plate at 4 °C (N = 6/group) and inserted into M2 Spectramax plate reader (Molecular Devices) pre-heated at 37 °C. Absorbance was measured at 405 nm every 15 s for 60 min, with the absorbance value corresponding to the degree of fibril density in the hydrogel. All values were normalized between 0 and 1 for each sample of tissue ECM by utilizing a ‘min-max’ normalization technique using Equation (2). Polymerization rate was calculated by taking the slope of the linear region of the curve.

(2) $$X^{\prime }=\left(X-\min\left(X\right)\right)/[\max\left(X\right)-\min\left(X\right)]$$

where X—set of absorbance values of x, X′—normalized value of X, min (X)—minimum value in X, max (X)—maximum value in X.

### 2.7. Mechanical Assessment of Human Tissue ECM Hydrogels

Mechanical assessment of tissue ECM hydrogels was conducted by performing uniaxial compression testing using a Discovery Series HR-30 Rheometer (TA, Instruments, New Castle, DE, USA) (N = 12/group). Briefly, the hydrogels were submerged in ultrapure water for 1 min, loaded onto a 20 mm sandblasted stainless-steel platform, and blotted carefully using a KimWipe to remove excess surface water. Following this, the hydrogels were compressed using a parallel plate geometry calibrated in DMA mode at a constant loading rate of 10 µm/s until 60% strain was achieved. Stress versus strain curves were generated for all the hydrogels and the compressive modulus was calculated by taking the slope between the 5–15% strain region [30].

### 2.8. Assessment of Tissue ECM Hydrogel Stability Using In Vitro Collagenase Assay

The stability of tissue ECM hydrogels was assessed via enzymatic degradation studies using an in vitro collagenase assay (N = 4/group). Briefly, the initial wet weight of the hydrogels (W_0_) was recorded by placing the hydrogels on a weighing scale. The hydrogels were then incubated in 500 µL of collagenase solution (5 U/mL in 0.1 M Tris-HCl buffer and 5 mM CaCl_2_; pH 7.4) at 37 °C in individual microcentrifuge tubes under constant stirring. At the 4 h timepoint, the hydrogels were blotted using a KimWipe to remove the excess water and weighed (W_t_). The residual mass of the hydrogels was calculated using Equation (3).

(3) $$Residual\;Mass\left(\%\right)=\frac{W_{t}}{W_{0}}\;x100$$

### 2.9. Assessment of Surface Morphology of Human Tissue ECM Hydrogels Using Scanning Electron Microscopy (SEM)

The surface microstructure of tissue ECM hydrogels was assessed using SEM (N = 6/group). Hydrogels were fixed in 2.5% glutaraldehyde solution (Cat# A17876.AE) (in 1x PBS) for 15 min and washed in ultrapure water. Following this, the hydrogels were dehydrated serially using ethanol (20%, 50%, 75%, 90%, and 100%) for 15 min at each concentration. Hydrogels were then submerged in 100% ethanol for 1 h and exposed to critical point drying (Leica EM CPD3000, Wetzlar, Germany). The dried hydrogels were placed on stubs, sputter coated with gold for 60 s, and imaged using SEM (Joel JSM-6380LV).

### 2.10. Cell Culture and Encapsulation in Human Tissue ECM Hydrogels

CMSC were maintained in growth medium composed of alpha Minimum Essential Medium (α-MEM) supplemented with 10% fetal bovine serum (FBS) and 1% penicillin/streptomycin in 75 cm^2^ culture flasks at 37 °C and 5% CO_2_. All experiments were conducted using passage 4 cells.

For 2D culture, 100 µL of tissue ECM solutions (0.1 mg/mL) were added to the 96-well plates and incubated at 37 °C for 2 h. Post incubation, the excess ECM solution was removed from each well, plates were washed with 1x PBS, and CMSCs were seeded at a density of 2000 cells/cm^2^. Cells were cultured in growth medium for 7 days and the media was refreshed every 3 days. For 3D culture, CSCs were suspended in a neutralized tissue ECM solution (10,000 cells/mL), added to a 96-well plate and incubated at 37 °C for 60 min to induce gelation. After gelation, hydrogels were cultured in growth medium for 7 days, and the media was replaced every 3 days.

### 2.11. Cell Viability, Metabolic Activity and Morphology in 2D Tissue Culture and 3D Cell-Laden Human Tissue ECM Hydrogels

Cell viability assessment in 2D culture and 3D hydrogels was performed using live-dead assay (N = 6/group/timepoint). At day 1 and day 7, α-MEM was aspirated from each well and the cells were washed with 1x PBS and stained with ethidium homodimer and calcein AM for 30 min at 37 °C. Following this, the stained cells were imaged under a fluorescence microscope (Olympus) to qualitatively assess cell viability. Cell metabolic activity was assessed by performing an alamarBlue^TM^ assay (N = 6/group/timepoint). At days 1, 4, and 7, cells were incubated in 10% alamarBlue^TM^ solution in α-MEM at 37 °C for 4 h. Following this, 100 µL aliquots from each well were transferred to a new 96-well plate and fluorescence was measured at an excitation wavelength of 555 nm and an emission wavelength of 595 nm using an M2 Spectramax plate reader. Relative fluorescence units (RFU) were reported as a measure of cell metabolic activity.

### 2.12. Rheological Characterization and Drop-On-Demand Printing of Human Tissue ECM Inks

Rheological properties of the tissue ECM inks (N = 3/group) were measured using a Discover Series HR30 Rheometer (TA Instruments, New Castle, DE, USA) to assess the shear thinning properties. Briefly, neutralized inks were loaded on to a 20 mm sandblasted steel platform and the shear stress and apparent viscosity was measured over a shear rate of 0.01–100 s^−1^. A logarithmic plot of apparent viscosity vs. shear rate was generated and the slope of the individual curves was determined to calculate the power law index (n). At zero shear, the consistency of the inks (K) was given by the y-intercept of the curve. The viscosity (η) was calculated at a shear rate (γ) of 10 s^−1^ by using Equation (4).

(4) $$\eta ={K\gamma }^{n-1}\;\;\;$$

Print fidelity of the tissue ECM inks (N = 4/group) was assessed by performing drop-on-demand printing using the CELLINK Bio-X6 3D printer (Biotechnology Company; Gothenburg, Sweden). Individual inks were neutralized in a syringe and loaded into printing cartridges. Drop-on-demand printing for 1 s at a pressure of 45 kPa was performed. High resolution images of the printed droplets were taken from a fixed distance, and droplet area was calculated using ImageJ (NIH; Bethesda, MD, USA).

### 2.13. Statistical Analysis

Results are expressed as mean ± standard deviation. Statistical analysis was performed using one-way ANOVA with Tukey post hoc for pairwise comparisons (JMP Pro 14 Statistical Discovery, SAS, Cary, NC, USA). Statistical significance was set at *p* < 0.05.

## 3. Results

### 3.1. Assessment of Decellularization Efficacy and Collagen Content in Different Human Tissue ECM

**Figure 1:**
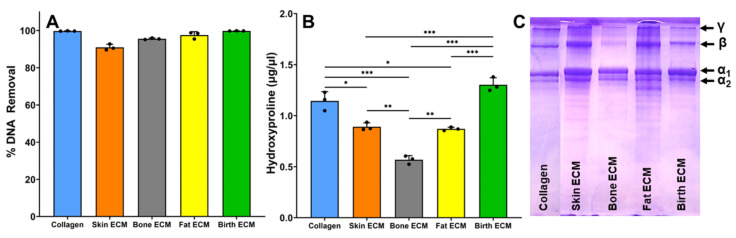
(**A**) Assessment of decellularization efficacy by quantification of % DNA removal from human-derived tissue ECMs, (**B**) Hydroxyproline content in different human-derived tissue ECM, (**C**) SDS Page showing bands for different molecular weights and presence of common collagen bands. (* indicates *p* < 0.05, ** indicates *p* < 0.01, *** indicates *p* < 0.001).

Assessment of decellularization efficacy was performed by quantifying the amount of remnant DNA post-decellularization and comparing the measurements with the total DNA in the tissue prior to decellularization. Results showed that >90% DNA removal from the tissue post-decellularization for all tissue ECM confirming that the decellularization protocols employed were efficacious (Figure 1A). Next, hydroxyproline quantification assay was performed to quantify the total collagen content in each tissue ECM. Results revealed that the collagen content in birth ECM was comparable to the control (pure collagen), indicating that birth ECM is predominantly composed of collagen (Figure 1B). In addition, collagen content in skin ECM, bone ECM, and fat ECM were significantly lower (*p* < 0.05) than pure collagen and birth ECM, indicating the presence of other matrix components. When comparing the different ECM sources, the collagen content in skin ECM and fat ECM was found to be significantly higher than bone ECM (*p* < 0.05), but comparable to each other (*p* > 0.05).

The SDS-PAGE analysis of the tissue-specific ECM revealed distinct bands at various molecular weights (Figure 1C). The analysis showed the presence of γ, β, α_1_ and α_2_ collagen chains for all tissue ECMs. There were some variations in the band patterns among the different tissue ECM, reflecting the unique composition of each tissue’s ECM. The purity of the extracted collagen type I was confirmed by the absence of additional bands or smearing in the gel, indicating minimal contamination by other proteins. Together, these results confirm the efficiency of the decellularization process and suggest possible variations in the compositional make up of ECM derived from different tissues.

### 3.2. Quantification of Gelation Kinetics of Different Human Tissue ECM Solutions

**Figure 2:**
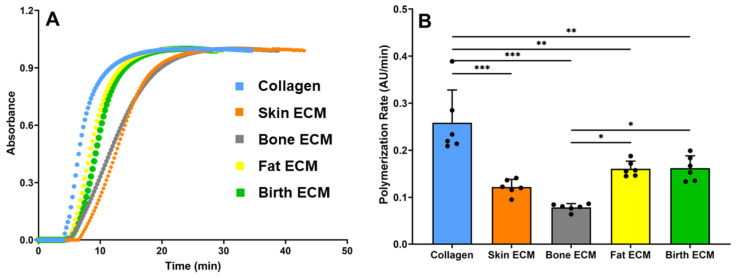
(**A**) Gelation kinetics, (**B**) Polymerization rate of tissue ECM (* indicates *p* < 0.05, ** indicates *p* < 0.01, *** indicates *p* < 0.001).

Results from the gelation kinetics assay showed the typical sigmoidal shape for all the tissue ECM hydrogels, which is indicative of the collagen fibrillogenesis process (Figure 2A). Expectedly, the polymerization rate of pure collagen was significantly faster compared to ECM derived from different tissues, suggesting that these ECM solutions comprise blends of collagen and other matrix proteins (Figure 2B). When comparing the different tissue ECM solutions, the polymerization rate of skin, fat, and birth ECM were comparable to, but significantly higher than, bone ECM (*p* < 0.05) indicating that compositional variations in ECM derived from different tissues significantly impact the gelation kinetics of hydrogels.

### 3.3. Mechanical Assessment and Stability of Human Tissue ECM Hydrogels

**Figure 3:**
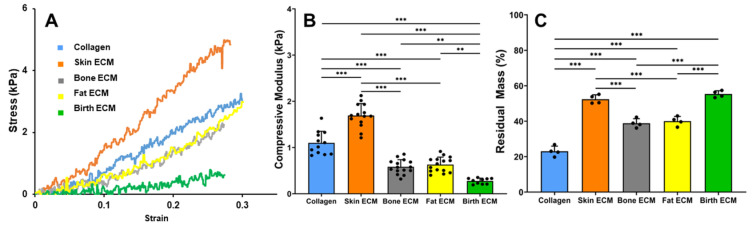
(**A**) Representative stress vs. strain curves, (**B**) Compressive modulus (**C**) Residual mass from in vitro collagenase degradation assay. (** indicates *p* < 0.01, *** indicates *p* <  0.001).

Uniaxial compression testing was performed to compare the compressive modulus of tissue ECM hydrogels. Stress vs. strain curves for each of the ECMs are shown in Figure 3A. The compressive modulus of pure collagen hydrogels was found to be around 1.1 kPa (Figure 3B). Hydrogels prepared using skin ECM showed significantly higher (*p* < 0.05) modulus compared to the control as well as ones made with other tissue ECM sources. Compressive modulus of bone ECM and fat ECM hydrogels were comparable at around 0.5 kPa. Birth ECM hydrogels showed the lowest compressive modulus at about 0.2 kPa, which was significantly lower (*p* < 0.05) than all other groups.

The stability of ECM hydrogels was assessed using an in vitro degradation assay to determine the residual mass of the hydrogels 4 h post incubation in collagenase solution. Pure collagen hydrogels degraded the fastest with only 20% residual mass at the 4 h timepoint (Figure 3C). The residual mass of skin and birth ECM hydrogels was comparable at around 50% and significantly higher (*p* < 0.05) than bone and skin ECM hydrogels. Together, these results indicate that the mechanical properties and stability of skin ECM hydrogels are significantly higher than other tissue-derived ECM hydrogels.

### 3.4. Surface Microstructure of Human Tissue ECM Hydrogels

**Figure 4:**
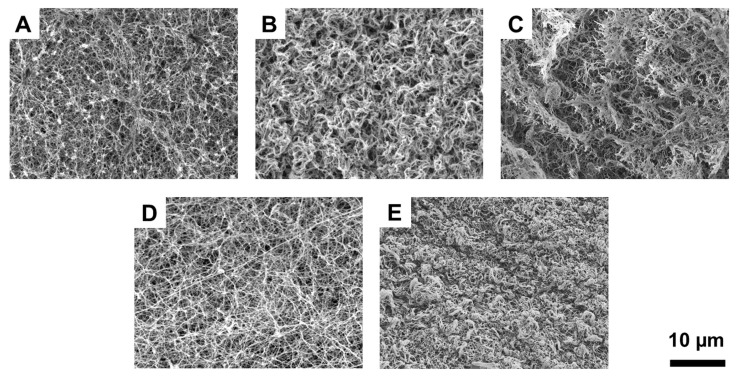
Surface microstructure of human-derived tissue ECMs–(**A**) Collagen, (**B**) Skin ECM, (**C**) Bone ECM, (**D**) Fat ECM, and (**E**) Birth ECM.

The surface morphology of the tissue ECM hydrogels was compared by performing SEM (Figure 4). Hydrogels fabricated using pure collagen and fat ECM showed a typical fibrous microstructure with randomly oriented collagen fibers (Figure 4A,D). Birth ECM hydrogels displayed a fibrous microstructure with highly dense packing of collagen fibers (Figure 4E). Similar surface microstructure was also observed for skin ECM hydrogels, albeit with visibly less dense packing of fibers and larger pore size compared to birth ECM hydrogels (Figure 4B). Hydrogels made using bone ECM showed some evidence of porosity along with a scaly surface topography (Figure 4C). In summary, SEM imaging revealed substantial differences in surface microstructure of hydrogels prepared using ECM solutions derived from different tissues.

### 3.5. Cell Viability and Metabolic Activity in 2D and 3D Culture Systems

**Figure 5:**
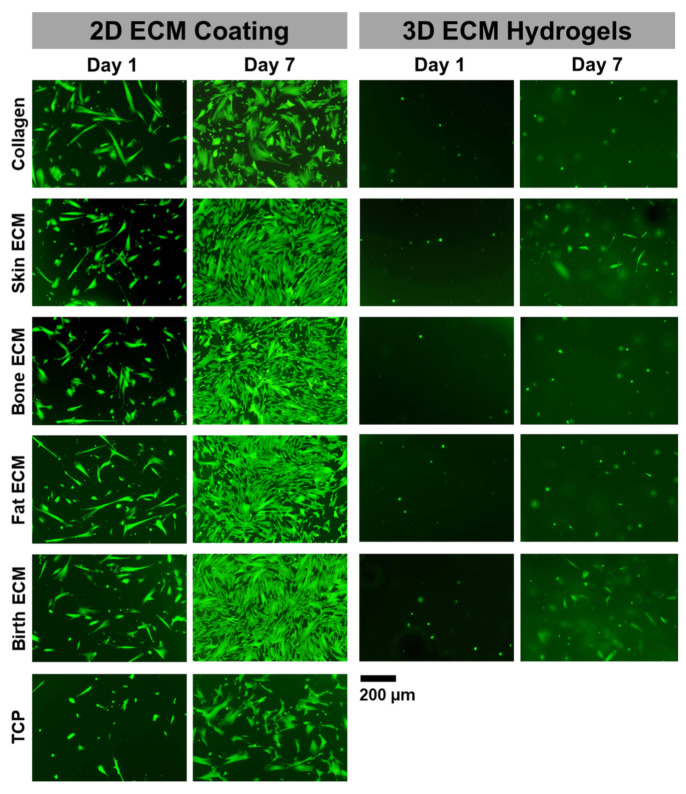
Assessment of cell viability using live-dead assay for cells cultured on 2D films and 3D hydrogels at days 1 and 7.

Cytocompatibility of CMSCs seeded on different ECM-coated tissue culture plates and CMSCs encapsulated within tissue-specific ECM hydrogels was evaluated using live-dead assay at day 1 and day 7. Results depicted excellent cell viability on day 1 for all the tissue-specific ECM-coated plates in 2D culture (Figure 5). Further, the cells displayed a well-spread morphology on day 7 and maintained their viability over time. CMSCs encapsulated within tissue-specific ECM hydrogels also showed high cell viability on day 1 and day 7.

**Figure 6:**
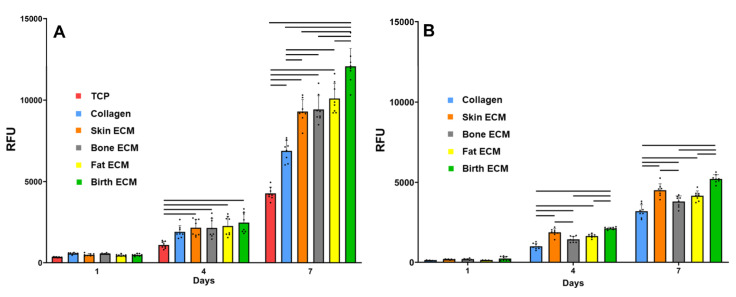
Assessment of cell metabolic activity using alamarBlue**^TM^** assay for cells cultured on (**A**) 2D films and (**B**) 3D hydrogels at days 1 and 7. (lines connecting groups indicate significant significance (*p* < 0.05)).

alamarBlue^TM^ assay was carried out to assess the cell metabolic activity in 2D and 3D culture systems over a period of 7 days. In the 2D culture system, the cell metabolic activity was comparable in all tissue ECM-coated plates on day 1 and increased significantly over time (Figure 6A). When comparing different tissue ECM-coated plates on days 4 and 7, cells seeded on coated plates showed significantly higher cell metabolic activity than uncoated tissue culture polystyrene (TCP). Cell metabolic activity at day 4 on all tissue-specific ECM-coated plates was comparable. However, at day 7, the metabolic activity of cells on plates coated with different tissue ECM outperformed the pure collagen coated plates. In addition, cell metabolic activity on birth ECM-coated plates was significantly higher (*p* < 0.05) than skin, bone, and fat ECM-coated plates.

A similar trend was seen for the 3D culture system wherein the cell metabolic activity was comparable in all hydrogels on day 1 and increased significantly over time (Figure 6B). At days 4 and 7, the cell metabolic activity in pure collagen hydrogels was significantly lower than tissue ECM hydrogels. Additionally, cells in birth ECM hydrogels showed significantly higher (*p* < 0.05) metabolic activity than bone and fat ECM hydrogels at both day 4 and day 7. Moreover, the cell metabolic activity in skin ECM hydrogels was significantly higher (*p* < 0.05) than bone ECM hydrogels at day 4 and day 7. Overall, these results suggest that metabolic activity of cells cultured in 2D and 3D pure collagen systems was lower than tissue-ECM-derived materials, indicating that a multicomponent ECM matrix can better support cell response.

### 3.6. Rheological Characterization and Printability of Tissue ECM Hydrogels

**Figure 7:**
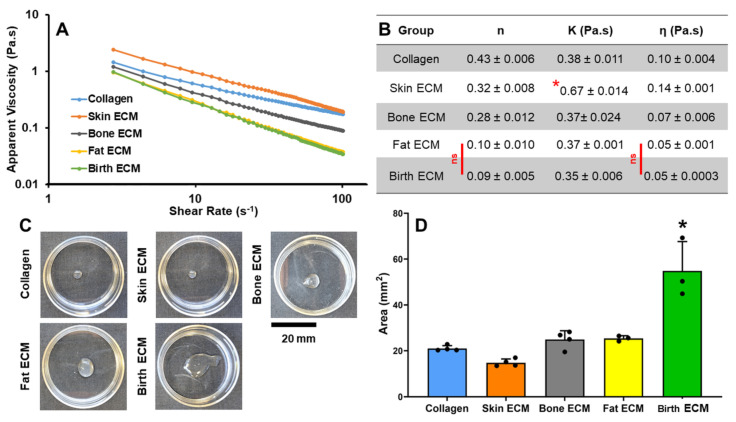
(**A**) Apparent viscosity vs. shear rate of different human-derived tissue ECM (**B**), Table showing differences in power law index, consistency, and viscosity. For power law index and viscosity data, groups connected by ‘ns’ indicate *p* > 0.05, all other comparisons are statistically significant (*p* < 0.05). Consistency data showed skin ECM was significantly higher (*p* < 0.05) than all other ECM (indicated by ‘*’), (**C**) Drop-on-demand printing technique showing differences in drop sizes of different tissue ECM inks. (**D**) Average area of drop sizes of prints of tissue ECM inks. (* indicates statistical significance (*p* < 0.05) compared to all other groups).

Rheological characteristics of ECM inks were evaluated by studying the change in apparent viscosity of the inks with varying shear rate. Pure collagen ink and inks from all tissue ECM sources showed shear thinning properties as indicated by decrease in viscosity with increase in shear rate confirming that these materials are suitable to be used as inks in extrusion-based 3D bioprinting (Figure 7A). The power law index of pure collagen ink was 0.43 and was significantly higher (*p* < 0.05) than all other inks (Figure 7B). The consistency of collagen, bone, fat and birth ECM inks were comparable, while skin ECM showed a significantly higher (*p* < 0.05) consistency of 0.67 Pa.s. The viscosity of pure collagen and skin ECM inks was significantly higher (*p* < 0.05) than bone, fat and birth ECM inks. Printability of tissue ECM inks was evaluated using a drop-on-demand printing technique, and the area of the printed drops was calculated for each of the tissue ECM inks (Figure 7C,D).

Comparable drop sizes were obtained for collagen control and skin ECM. Bone and fat ECM inks also showed similar drop sizes. Prints obtained from birth ECM showed the largest drop size with an area of drop significantly higher (*p* < 0.05) than all other tissue ECM inks. Combined results from rheological characterization and drop-on-demand printing revealed that skin ECM exhibits higher viscosity and superior printability compared to other ECMs, indicating that compositional differences in tissue-specific ECMs may influence the use of these materials as bioinks in 3D bioprinting applications.

## 4. Discussion

Human-derived ECMs possess a unique composition that closely resembles the native tissue ECM, and can therefore provide a biologically relevant environment for the cells. Native human ECM composition includes essential matrix proteins and growth factors that play a critical role in cellular signaling, adhesion, and tissue remodeling [8]. While synthetic hydrogels may offer better control of physical properties, they often lack the intricate biochemical cues necessary for optimal cell behavior and tissue regeneration [31]. Therefore, the use of human-derived ECMs may be a preferred choice in tissue engineering and regenerative medicine applications as they provide a biologically compatible substrate for supporting cellular and tissue responses.

Successful removal of cellular components while preserving the structural proteins of the ECM is crucial for maintaining the biological and functional properties of ECM-derived hydrogels [32]. The efficient decellularization process, as evidenced by over 90% DNA removal (Figure 1A), is consistent with previous studies that employed similar decellularization protocols [33,34]. Quantification of collagen content using the hydroxyproline assay revealed noteworthy differences among the tissue ECMs (Figure 1B). Birth ECM exhibited a collagen content comparable to pure collagen control, suggesting that birth ECM is predominantly composed of collagen. This finding is consistent with previous research demonstrating the high collagen content in birth-derived ECMs [35,36]. In contrast, skin, bone, and fat ECMs showed significantly lower collagen content, indicating the presence of other matrix proteins. For example, bone ECM comprises collagen types I, III, and V, as well as non-collagenous proteins, like osteopontin and osteocalcin, which play critical roles in bone formation and mineralization [37]. The characterization of tissue-specific ECMs through SDS-PAGE analysis confirmed the presence of distinct bands at various molecular weights for the tissue ECM hydrogels (Figure 1C), indicating the presence of different protein components within the ECM, as observed in previous studies [36,38,39]. These bands corresponded to the γ, β, α_1_, and α_2_ collagen chains, which are characteristic of collagen type I, the main component of the ECM. The presence of these collagen chains confirmed the integrity of the tissue-specific ECM hydrogels. In addition to collagen, other low molecular weight proteins and peptides may be present in the ECM hydrogels, as observed in this study. This knowledge is essential for understanding the molecular characteristics of ECM hydrogels and their potential influence on cellular behavior and tissue regeneration. For instance, skin ECM may contain additional proteins, like elastin, laminin, and fibronectin, which contribute to the mechanical and elastic properties of the skin [10]. These findings align with the existing knowledge that different tissues possess diverse ECM compositions due to the presence of additional matrix components, such as proteoglycans, glycosaminoglycans (GAGs), and glycoproteins [26,40].

The gelation kinetics assay demonstrated sigmoidal gelation profiles for tissue ECM hydrogels, indicating collagen fibrillogenesis (Figure 2A). Pure collagen exhibited a faster polymerization rate compared to tissue-specific ECMs. Previous studies on ECM-derived hydrogels from different sources have also reported a delay in fibrillogenesis, similar to the findings presented in this study. For example, hydrogels derived from demineralized and decellularized bone exhibited a lag phase of approximately 9 min [41]. Sackett et al. observed similar results where the gelation speed of human-pancreas-derived ECM hydrogel was slower than pure collagen hydrogel [42]. Birth ECM, despite having a comparable hydroxyproline content to collagen, contains different types of collagens (III, IV, VII, and XVII) and other non-collagenous proteins that could interact with collagen type I molecules, affecting their crosslinking and subsequent gelation kinetics [43,44]. Furthermore, when comparing the different tissue ECM solutions, the polymerization rates of skin, fat, and birth ECM were comparable to each other but significantly higher than bone ECM (Figure 2B). Slower gelation kinetics of ECM hydrogels can be beneficial for tissue-specific applications to allow for better cell encapsulation, enhanced drug and growth factor delivery, and improved integration with the surrounding tissue [45]. Further, longer polymerization time can minimize the risk of clogging the extrusion tip for 3D bioprinting of large tissue scaffolds. These observations indicate that the time required for the initiation of collagen fibrillogenesis can vary depending on the tissue source and the specific composition of the ECM.

Mechanical assessment of human-derived tissue ECMs revealed that pure collagen and skin ECM hydrogels showed a significantly higher compressive modulus compared to bone, fat, and birth ECM hydrogels (Figure 3A,B). The higher compressive modulus of skin ECM hydrogels can be attributed to the rich presence of classical fibrillar collagens (type I, II, III, V, and XI) in skin ECM as opposed to that found in ECM of other human tissues, which can enable the formation of more robust intrafibrillar crosslinks [46,47]. In addition, interactions of specific tissue ECM components, such as elastin, laminin, decorin, and chondroitin sulfate, with collagen contribute to the maintenance of tissue homeostasis and the preservation of mechanical integrity in skin ECM hydrogels. Although collagen type I is predominant in most tissue ECMs, non-fibrillar collagen type IV and type VI are largely present in birth and fat ECM, respectively, which could explain the lower compressive modulus of those hydrogels [48,49]. Further, the modulus of bone ECM was found to be comparatively weaker than the other tissue ECMs because bone ECM was subjected to a process of demineralization prior to extraction. By removing the mineral content, the resulting bone ECM reflects a diminished strength, highlighting the significance of minerals in conferring robustness to bone tissue [50]. In vitro collagenase assay for assessment of hydrogel stability showed that pure collagen hydrogels degrade most quickly, followed by bone and fat ECM hydrogels (Figure 3C). The presence of intrafibrillar collagen crosslinks in skin ECM hydrogels can provide better resistance to enzymatic degradation, which is evident from the significantly higher residual mass for skin ECM hydrogels at the 4-h timepoint. Higher stability of skin and birth ECM can also be explained by the higher collagen content as observed from the results of the hydroxyproline assay. Additionally, longer degradation times of skin and birth ECM hydrogels can be due to the denser packing of collagen fibers in these hydrogels compared to bone and fat ECM hydrogels (Figure 4).

Results from cell studies showed that ECM coatings and hydrogels support CMSC viability (Figure 5). Application of ECM coating onto TCP significantly enhances cell proliferation (Figure 6A). A similar outcome was also observed on 3D hydrogels, wherein cell metabolic activity was significantly higher (*p* < 0.05) on ECM hydrogels compared to pure collagen ones (Figure 6B). When comparing the ECM hydrogels from different tissue sources, higher cell metabolic activity on skin and birth ECM hydrogels may be attributed to the greater collagen content, as shown by the hydroxyproline assay (Figure 1B). In addition, highly dense collagen fibers observed with SEM for birth and skin ECM (Figure 4) might allow for enhanced cell-matrix interactions and signaling, thereby promoting higher metabolic activity [51].

Extrusion-based 3D printing utilizes a shear thinning hydrogel-based ink with or without cells that is precisely deposited through a nozzle in a layer-by-layer fashion to fabricate complex biological structures [52]. While extrusion 3D printing has the potential to revolutionize the field of tissue engineering, it is necessary to address the challenges with ink optimization for tissue-specific applications. In this work, the rheological properties and drop-on-demand printability of unmodified ECM inks derived from different human tissues were assessed by employing a standard ink concentration of 6 mg/mL to facilitate comparison. However, considering the unique composition of each ECM, it is crucial to determine an appropriate concentration, printing parameters, and platform that ensures optimal printing of the tissue ECMs. Results revealed that skin ECM inks exhibited significantly higher (*p* < 0.05) apparent viscosity compared to other ECM inks, showcasing superior printability as indicated by smallest droplet size (Figure 7B). Bone ECM, fat ECM and pure collagen inks also exhibited shear thinning properties (Figure 7A) with printability comparable to that of skin ECM (Figure 7C,D). The printability of birth ECM inks was inferior compared to other inks, which may be due to the lower apparent viscosity of birth ECM. One approach to improve the printability of birth ECM inks may be to modify the collagen backbone via methacrylation of the free amine groups, which can enable photocrosslinking of the printed construct for better retention of shape fidelity [53,54].

In conclusion, results from this study demonstrate that the physicochemical and biological properties of ECM hydrogels vary significantly depending on the tissue source. Specifically, bone ECM polymerizes significantly more slowly than ECM derived from skin, fat, and birth sources. The compressive modulus of skin ECM hydrogels was the highest and birth ECM hydrogels was the lowest. Birth ECM hydrogels showed significantly higher cell metabolic activity in both 2D and 3D environments, possibly due to higher total collagen content and highly dense fibers that better support cell growth. All ECM inks exhibited shear thinning properties with skin, bone, and fat ECM inks demonstrating better printability than birth ECM ink. Based on these outcomes, the tissue source must be considered as an important factor for the design and development of ECM scaffolds for tissue engineering and regenerative medicine therapies. 

## Data Availability

The data that support the findings of this study are available from the corresponding author upon reasonable request.
